# Analysis of B7-H4 expression in metastatic pleural adenocarcinoma and therapeutic potential of its antagonists

**DOI:** 10.1186/s12885-017-3615-8

**Published:** 2017-09-18

**Authors:** Cheng Chen, Qiu-Xia Qu, Fang Xie, Wei-Dong Zhu, Ye-Han Zhu, Jian-An Huang

**Affiliations:** 1 Respiratory Department, The First Affiliated Hospital of Soochow University, 188 Shizi Street, Suzhou, 215006 China; 2 Clinical Immunology Laboratory, The First Affiliated Hospital of Soochow University, 788 Renmin Road, Suzhou, 215007 China; 3 Pathology Division, Soochow University, 1 Shizi Street, Suzhou, 215006 China; 4 Pathology Department, The First Affiliated Hospital of Soochow University, 188 Shizi Street, Suzhou, 215006 China

**Keywords:** Metastatic pleural neoplasms, Adenocarcinoma, B7-H4, Immunotherapy

## Abstract

**Background:**

The increasing incidence and poor outcome associated with malignant pleural effusion (MPE) requires finding an effective treatment for this disease. Inhibitory B7-H4 is expressed in many different human cancers but its role in malignant pleural tissue has yet to be established.

**Methods:**

Here, patients with metastatic pleural adenocarcinoma (MPA) or with early-stage lung adenocarcinoma were clinically and statistically analyzed. Immunohistochemistry and confocal microscopy were used to determinate the expression of B7-H4 in the cancer cells. By using MPE model, we sought to a potential immunotherapy for MPE with anti-B7-H4 mAb.

**Results:**

When compared to early-stage lung adenocarcinoma, MPA possessed higher level of nuclei membranous B7-H4 and lower cytoplasmic B7-H4 expression. Also, nuclei membranous B7-H4 expression was found to be positively correlated to Ki-67 expression, and indicated a possible poor prognosis of MPA. In mouse MPE model, intra-pleurally injection of anti-B7-H4 mAb effectively suppressed MPE formation.

**Conclusions:**

Taken together, our data was in support of the significance of B7-H4 expression in MPA, which also suggest it warrants further exploration for potential immunotherapy of MPE.

## Background

Clinically, pleural effusion (PE) is a common condition caused by malignant tumors, as well as benign diseases [1]. For malignant pleural effusion (MPE), malignant cells would establish the way to the pleura through direct extension or through lymphatic or hematogenous spread [2, 3]. The reason for the aggressive behavior of malignant cells can be attributable to their molecular aberrations. So, the better knowledge of histopathological features and the available molecules could clinically been applied for diagnosis and therapy of MPE.

B7-H4, an cell surface immunomodulatory glycoprotein, was primarily found to express on antigen-presenting cells and in some non-hematopoietic tissues. It has also been demonstrated that B7-H4 is broadly expressed in many malignant tumors including carcinomas of the hepatocellular, lung, osteosarcoma and ovarian, and it contributes to the tumor immune escape [4–7]. It has also been reported that measurement of sB7-H4 might bel diagnostic value for MPE [8]. However, the B7-H4 expression in metastatic pleural adenocarcinoma (MPA) has not been widely reported. Furthermore, since PD-L1 and PD-1 mAb revolutionized cancer immunotherapy, an impressive variety of clinic trials of checkpoint blockade were already underway or planned [9]. At this stage, as another checkpoint, B7-H4 might also participate to tumor progression and be a candidate target to cancer immunotherapy [10, 11].

Also, the detection of B7-H4 in cytoplasm of tumor cells suggested it might have some unanticipated function that is different from membrane B7-H4. For example, Zhang L et al. revealed that B7-H4 promote renal cell carcinoma progression and cell proliferation through translocating into nucleus [12]. It was also suggested that the intracellular B7-H4 appears to prevent Fas/FasL-mediated bile duct epithelial cells apoptosis during the progression of primary biliary cirrhosis (PBC) [13].

In present study, we compared the expressing profile of B7-H4 in MPA to that in early-stage lung adenocarcinoma, and to determine whether B7-H4 could be used as a carcinogenic factor for MPE. Finally, by using MPE model, we sought to further investigate whether anti-B7-H4 mAb treatment could be used as a potential immunotherapy for MPE.

## Methods

### Cell line and mAb

Six to eight week-old female C57BL/6 mice (H-2^b^) were purchased from Chinese Academy of Sciences, Shanghai Institutes for Biological Sciences, Experimental Animal Center. Lewis lung carcinoma cell line (LLC) was purchased from Chinese Academy of Sciences, Shanghai Institutes for Biological Sciences. The cell was cultured in RPMI1640 supplemented with 10% fetal calf serum (FCS), 2 mM L-glutamine, 100 mg/mL streptomycin, 100 U/mL penicillin, and 50 mM 2-ME. The cell was incubated at 37 °C with 5% CO_2_.

### Patients

Twenty-three patients with PE were admitted at The First Affiliated Hospital of Soochow University from 2011 to 2016. All patients were proven histologically diagnosis of MPA by pleural biopsy under thoracoscope. The subjects also comprised 9 patients with solitary pulmonary nodules (SPN) who underwent thoracotomy or selected video-assisted thoracic surgery (VATS) between 2012 and 2015. Clinicopathologic information included complete history, age, sex and histology subtype.

### Immunohistochemistry

Formalin-fixed, paraffin-embedded samples were cut (4 μm-thick sections) and placed on silane-covered slides. Morphological assessment was obtained by hematoxylin-eosinsaffron staining. In brief, after dewaxing, inactivating endogenous peroxidase activity and blocking cross-reactivity with 3% BSA, all sections were incubated at 37 °C for 1 h with diluted solution of the B7-H4 mAb (ployclonal antibody, Novus) and Ki-67 mAb (Genetech, clone GM001), respectively. Location of the primary antibodies was achieved by subsequent reaction with a horseradish peroxidase-conjugated anti-primary antibody. Negative controls were established with mouse IgG (BD PharMingen) by replacing the primary antibody.

### Evaluation of immunohistochemical results

Five high-power fields were randomly selected. The score of the B7-H4 staining was categorized into five semi-quantitative classes based on the percentages of positive tumor cells: 0 (<5%), 1 (6–25%), 2 (26–50%), 3 (51–75%) and 4 (>75%). The intensity of cellular staining was also assessed semi-quantitatively on a scale of 0–3 as follows: 0 (negative), 1 (weakly positive), 2 (moderately positive) and 3 (strongly positive). The scores of intensity and the percentage gave rise to the final staining result: - (0), + (1–2), ++ (3–4), and +++ (5–7). During statistical analysis, tumors have a final result of - or +, which showed a no/low immunoreactivity, compared to tumors with result of ++ or +++ as the high immunoreactivity. The expression of Ki-67 was evaluated according to the percent of its staining and scored.

### Confocal microscopy

MPA sections were blocked with 3% BSA for 30 min and incubated for 1 h with PE-labeled anti-B7-H4 Ab. After washes, sections were incubated with 4,6-diamidino-2-phenylindole (DAPI, Roche Diagnostics). Fluorescence was visualized with Axiophot 1. Images were captured with an Axiocam color charge-coupled device camera and analyzed with AxioVision software (Carl Zeiss) [14].

### MPE model

Murine MPE models were prepared according to the methods described by Servais EL [15] by intrapleural injection of LLC cells (10^6^/mouse). Briefly, slowly insert the needle to the intercostal space between the diaphragm and lung at a shallow angle of 15°, needle bevel up, entering the pleural space through the diaphragm. At days 14 after intrapleural injection of LLC cells, all mice were performed CT scan to confirm the formation of pleural effusion. The CT images were captured with the mouse under anesthesia in a common animal holder.

### In vivo experiment

Mice with pleural effusion were received local injection of 50 μg anti-B7-H4 neutralized mAb (clone 9, eBioscience), or 50 μg mouse isotype mAb (eBioscience), respectively. After 7 days, CT scan was repeated.

### Statistical analysis

Statistical analysis was performed with SPSS statistical software (Version 19.0; SPSS Inc., Chicago, IL, USA). Overall survival (OS) was calculated from diagnosis of disease until the last follow-up for alive patients or until death due to any cause. OS analysis was carried out using the Kaplan-Meier curves [16]. The difference of B7-H4 expression in MPA and early-stage lung cancer was evaluated by using x^2^ tests. *P* values <0.05 were considered significant.

## Results

### Patient population

Baseline characteristics of all subjects were listed in Table 1. 23 patients underwent the pleural biopsy under endoscope and diagnosed by MPA. 9 patients underwent the surgery and diagnosed by stage I, which included 2 atypical adenomatous hyperplasia (AAH), 3 adenocarcinoma in situ (AIS), 4 lepidic adenocarcinoma (LA).

**Table 1:** B7-H4 expression in metastatic pleural adenocarcinoma

Parameter	Nuclear membrane					Cytoplasm		
	Low			High		Low		High
Age (MPN, median = 58)								
> 58		1		10		6		5
≤ 58		4		8		6		6
Age (LAC-stage I, median = 62)								
> 62		4		0		0		4
≤ 62		4		1		0		5
MPA								
Adenocarcinoma		5		18*		12		11**
﻿﻿LAC-Stage I		8		1		0		9
AAH		2		0		0		2
AIS		2		1		0		3
Lepidic		4		0		0		4

*P < 0.01 compared to the LAC-Stage I, **P = 0.012 compared to the LAC-Stage I. Metastatic pleural adenocarcinoma, MPA, LAC-stage I, lung adenocarcinoma-stage I

### B7-H4 expression in MPA

**Fig. 1:**
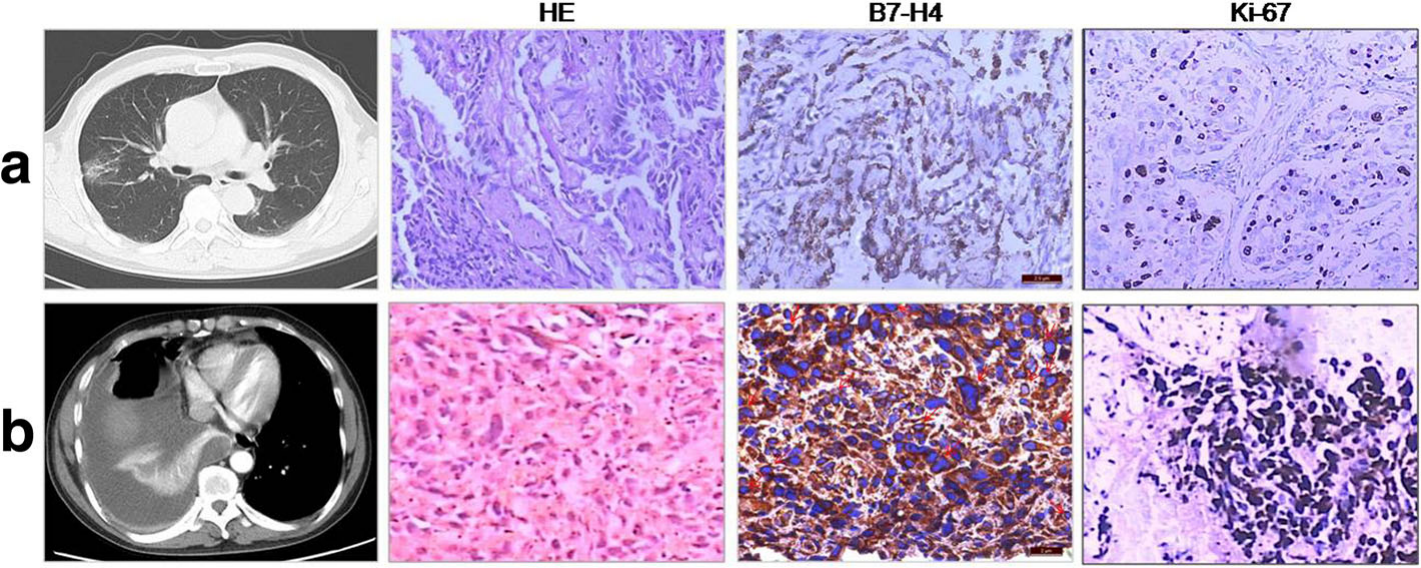
Immunostaining of B7-H4 and Ki-67 in lung adenocarcinoma. **a** line, CT scan shows opacity with ground-glass in the right lung, HE staining confirmed lepidic predominant adenocarcinoma with high differentiation, IHC demonstrated a negative nuclei membranous B7-H4 and low Ki-67 staning. **b** line, CT scan shows pleural effusion in the right lung, HE staining confirmed MPA, IHC demonstrated a high nuclei membranous B7-H4 and strong Ki-67 staning, (red arrow, ×40). One representative data was showed

**Fig. 2:**
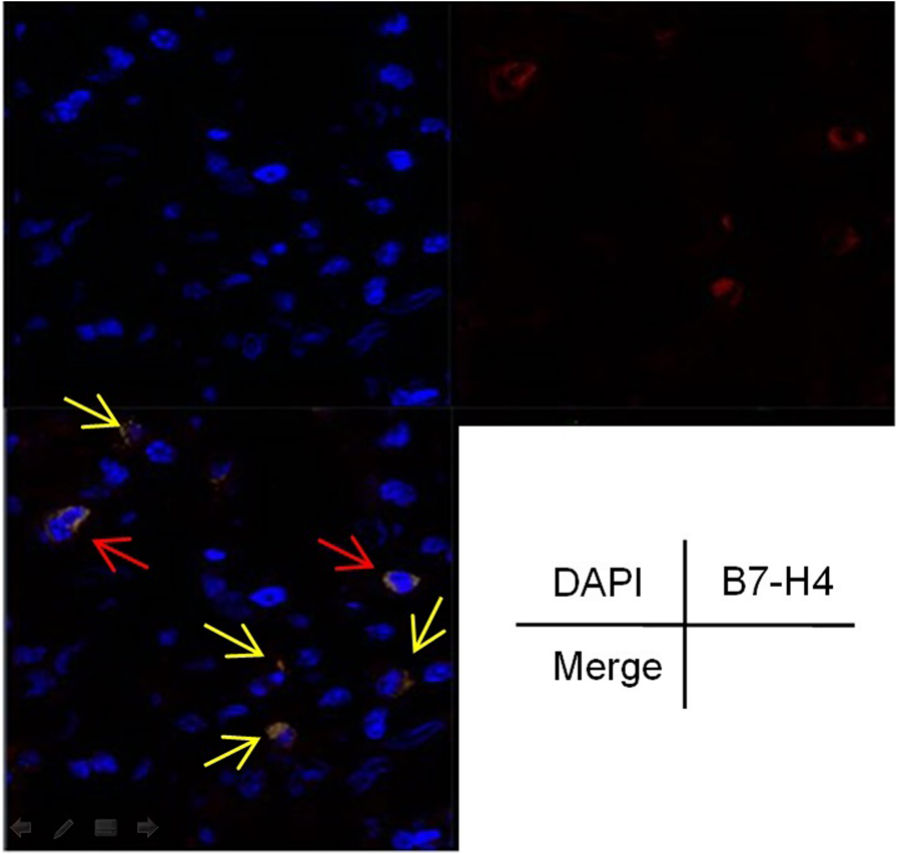
The expression of B7-H4 in the MPA was investigated by Confocal Microscopy. Images were captured with an Axiocam color charge-coupled device camera, one representative nuclei membranous B7-H4 (red arrow) and cytoplasmic B7-H4 (yellow arrow) was shown

**Fig. 3:**
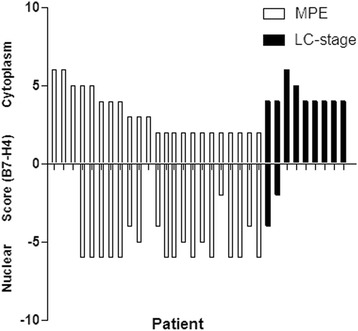
The overall view of cytoplasmic and nuclei membranous B7-H4 expression in the two groups of lung adenocarcinoma (MPA and LC-stage I)

B7-H4 was found to express along the nuclear membrane in 18 (78.3%) of 23 MPA by immunohistochemistry analysis. High cytoplasmic immunostaining of B7-H4 was found to be in 47.8% (11/23) cases (Table 1 and Figs. 1, 2, 3). Additionally, cytoplasmic B7-H4 and nuclei membranous B7-H4 immunostaining were also confirmed in situ by Confocal Microscopy (Fig. 2). Then, we used patients with AAH, AIS or LA as early-stage of lung cancer. Of note, when compared to MPA, early-stage of lung cancer possessed higher level of cytoplasmic B7-H4, and only rare cases (11.1%) were stained positively with nuclei membranous B7-H4 (Table 1 and Figs. 1, 3). Taken together, our data demonstrate a distinct B7-H4 expression between early-stage of lung adenocarcinoma and MPA, decrease of cytoplasmic and occurrence of nuclear membranous B7-H4 was associated with the increase of malignancy of cancer cells and development of MPA.

### Expression of B7-H4 and Ki-67 in MPA

**Fig. 4:**
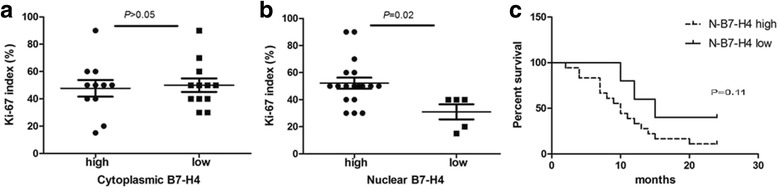
Correlation of the Ki-67 index with cytoplasmic (**a**) and nuclei membranous (**b**) B7-H4 in MPA patients was shown respectively. Kaplan-Meier survival curves for MPA patients according to expression level of nuclei membranous B7-H4 was shown in (**c**)

Furthermore, we also assessed expression of Ki-67, an identified proliferation antigen of the carcinomas, to explore whether B7-H4 expression is associated with increased cancer cell proliferation. As shown in Fig. 4, Ki-67 immunostaining was correlated to nuclei membranous B7-H4 (*P* < 0.05), but not to its expression in cytoplasm (*P* > 0.05), which suggested that nuclei membranous B7-H4 may be regarded as a proliferative factor for MPA.

### Impact of B7-H4 expression on survival of MPA

Additionally, we analyzed the outcomes for patient’s overall survival according to B7-H4 staining patterns. For B7-H4 expression, patients were grouped as “high” or “low” using the nuclei membranous immunostaining. 23 cases had follow-ups for 24 months for observing OS (Fig. 4c). Median survival for high nuclei membranous B7-H4 patients was 10 months and 15 months for B7-H4 low patients, indicating that nuclei membranous B7-H4 expression has possible impacts on survival of MPA patients. Due to low patient’ number here, it would attach statistical significance if we expanded the sample size.

### Efficacy of B7-H4 mAb on malignant pleural effusion

**Fig. 5:**
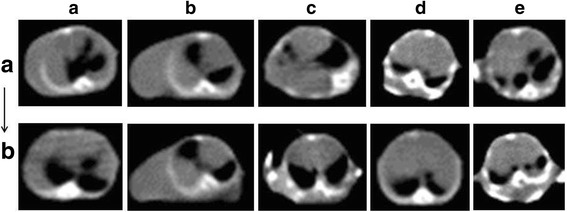
The overall view of MPE in the five mice was shown. **a**, before anti-B7-H4 mAb injection, **b**, after anti-B7-H4 mAb injection

To determine whether B7-H4 expression affects formation of MPE, we tested the volume of MPE by CT scan in MPE mice before and after anti-B7-H4 mAb treatment. At the same slice, we can semi-quantitative the MPE by fluid sonolucent area noticed in mediastinal window. We showed that the intra-pleurally anti-B7-H4 mAb treatment significantly suppressed MPE (Fig. 5).

## Discussion

B7-H4 has been found to be expressed at both mRNA and protein levels in many types of cancers, and the negative clinical associations of B7-H4 was found in lung, kidney, prostate, and gastrointestinal cancers. B7-H4 deficiency in mice resulted in significant protection from lung metastases and increased survival in the 4T1 tumor model. Mechanically, B7-H4 prompted metastasizing cancer cells to escape local antitumor immune responses through interactions with the innate and adaptive immune cell [17]. However, some studies also showed a role for B7-H4 in enhancing antitumor immunity. Clinically, breast cancer patients with increased B7-H4 expression showed a prolonged time to recurrence [18]. It was also demonstrated an effective immune regulation due to the B7-H3, but not the B7-H4 in the cancer microenvironment [19]. Given this unexpected phenotype, we extended our analysis of B7-H4 in MPA.

Here, 78.3% and 47.8% of MPA tissues were found to express nuclear membranous and cytoplasmic B7-H4 respectively. When using early-stage lung cancer as control, it is indicated that there was a distinct B7-H4 expression possessed by MPA. In brief, it was noted that the almost all AAH, AIS and LA were absent in expression of B7-H4 by nuclear membrane, instead of high cytoplasmic staining. In according to our data, we hypothesized that B7-H4 might work as a nuclear shuttling protein along with tumor progress, which mean poorer differentiated and more invasive adenocarcinoma would have higher nuclear membranous B7-H4 expression.

As previously reported, in immune-deficient mice, over-expression of B7-H4 could promote tumorigenesis of ovarian cancer by increased proliferation, adhesion, migration and invasion. In others’ study, over-expression of B7-H4 on epithelial cells could result in malignant cellular transformation, perhaps though protecting the pre-transformed cells from apoptosis. So, it implied that B7-H4 might have a direct effect on tumorigenesis independent of immune property [12, 13, 20]. As supported, our data indicated that nuclei membranous B7-H4 has a positive correlation to the expression of Ki-67, and it might have a functional activity in promoting cancer cell growth.

In the last decade, immune checkpoint inhibition had led to major therapeutic advances in tumor oncology. Given that B7-H4 is widely expressed in all examined cancer specimens and its inhibitory immune function, this paradigm called for the development of novel cancer immunotherapy strategies by targeting B7-H4. Taking advantage of an orthotopic model that faithfully mimics human pleural malignancy, we evaluated administration of B7-H4 mAb treatment to MPE. As expected, it was found that intra-pleurally administered B7-H4 mAb could decrease MPE production. It was supported by Adusumilli’s study, which demonstrated that intra-pleurally administered chimeric antigen receptor (CAR)-engineered T cells induce long-term complete remissions in malignant pleural mesothelioma (MPM) model [21]. Also, immune checkpoint inhibitors have been linked to the development of certain adverse events, such as cutaneous, hepatic and gastrointestinal toxicities, which commonly described as immune-related adverse events (irAEs) [22–24]. We believed that the routine of intra-pleurally administered B7-H4 mAb will be utilized in clinical studies, especially can decrease the risk of irAEs induced by systemic administration.

## Conclusions

Taken together, in the present study, our data in support of the functional role of B7-H4 in MPA come from evidence of B7-H4 expression on cancer cells and therapeutic efficacy of B7-H4 mAb on MPE. We believe that B7-H4 could become a valuable tool to add to the oncologist’s toolbox for predicting the prognosis of MPA patients. Although patient’ number was relatively low here, it has deepen our knowledge about biomarkers for analysis of the occurrence of MPA. This may be possible when B7-H4 was investigated on a larger number of cases in the future. Consequently, B7-H4 blockade could also offer a new therapeutic strategy for MPE [25].

## Abbreviations

AAH: atypical adenomatous hyperplasia
AIS: adenocarcinoma in situ
B7-H4: B7 homlogs 4
CAR: chimeric antigen receptor
DAPI: 4,6-diamidino-2-phenylindole
IrAEs: immune-related adverse events
LA: lepidic adenocarcinoma
Mc Ab: monoclonal antibody
MPM: malignant pleural mesothelioma
PE: pleural effusion
